# CRMP4-Mediated Regulation of Cav1.3 Protein Stability and Apoptosis Resistance in Endometriosis

**DOI:** 10.3390/ijms27209291

**Published:** 2026-10-19

**Authors:** Yi Huang, Yidan Wang, Ruiyun Li, Yuan Yang

**Affiliations:** 1 The First Clinical Medical College, Lanzhou University, Lanzhou 730000, China; 2 The Reproductive Medicine Center, The First Hospital of Lanzhou University, Lanzhou 730000, China; 3 Base of Reproductive Medicine Transformation Application, Key Laboratory for Reproductive Medicine and Embryo, Gansu International Scientific and Technological Cooperation, Lanzhou 730000, China; 4 Department of Obstetrics and Gynecology, The First Hospital of Lanzhou University, Lanzhou 730000, China

**Keywords:** endometriosis, apoptosis, ubiquitination, Cav1.3, CRMP4, ZNF460

## Abstract

Endometriosis (EMs) is a common chronic gynecological disorder characterized by the ectopic implantation and growth of endometrial-like tissue. The abnormal survival of ectopic endometrial cells, including their reduced susceptibility to apoptosis, plays an important role in the development of EMs. Cav1.3 is aberrantly upregulated in EMs and contributes to disease progression; however, the mechanism responsible for its increased expression remains unclear. In this study, protein stability and ubiquitination assays were performed in hEM15A cells. Cav1.3-interacting proteins were screened by co-immunoprecipitation coupled with liquid chromatography tandem mass spectrometry, and the regulatory pathway was further examined using in vitro experiments and animal models. We found that reduced ubiquitin–proteasome-mediated degradation increased Cav1.3 stability in hEM15A cells. Our results further showed that ZNF460 increased CRMP4 expression by activating its transcription. CRMP4 associated with Cav1.3 and reduced RNF138–Cav1.3 association, accompanied by decreased Cav1.3 ubiquitination and increased protein stability. ZNF460-dependent CRMP4 expression promoted Cav1.3 accumulation and was associated with reduced apoptosis in hEM15A cells. These findings clarify the post-translational mechanism underlying Cav1.3 upregulation in EMs and suggest that the ZNF460/CRMP4/RNF138/Cav1.3 axis may serve as a potential molecular target for EMs treatment.

## 1. Introduction

Endometriosis (EMs) is a common estrogen-dependent chronic inflammatory disease characterized by the implantation and growth of endometrial-like tissues outside the uterine cavity, which can lead to clinical manifestations such as chronic pelvic pain, dysmenorrhea, and infertility, significantly affecting the quality of life of reproductive-aged women [1,2]. Although the retrograde menstruation theory proposed by Sampson provides a widely accepted explanation for the origin of ectopic endometrial tissues, approximately 90% of women experience retrograde menstrual flow, whereas only a small proportion develop clinically apparent EMs, indicating that additional mechanisms determine the survival and persistence of ectopic endometrial cells [3]. Increasing evidence suggests that resistance to apoptosis, enhanced proliferation, inflammatory responses, and abnormal estrogen signaling contribute to the establishment and maintenance of ectopic lesions [4,5]. However, the molecular mechanisms responsible for apoptosis resistance in ectopic endometrial cells remain incompletely understood.

Voltage-dependent L-type calcium channel Cav1.3 is an important transmembrane protein involved in calcium influx and intracellular calcium homeostasis regulation. Cav1.3 plays essential roles in excitable tissues, including the nervous system, cardiovascular system, and endocrine organs [6]. Recent studies have demonstrated that dysregulated Cav1.3 expression is also associated with tumor progression by promoting cell proliferation, migration, and survival through calcium-dependent signaling pathways [7,8]. In prostate cancer, increased Cav1.3 expression contributes to androgen-dependent tumor growth, whereas in endometrial cancer, estrogen signaling promotes Cav1.3 expression and activates downstream ERK1/2-CREB pathways to enhance malignant biological behaviors [9,10]. Our previous study demonstrated that Cav1.3 is significantly upregulated in ectopic endometriotic lesions and endometriosis-derived stromal cells, where it suppresses apoptosis by reducing PARP cleavage and caspase-3 activation. However, the molecular mechanisms responsible for the abnormal accumulation and stabilization of Cav1.3 in EMs remain unknown.

Protein homeostasis is precisely regulated at both transcriptional and post-translational levels. Among various post-translational regulatory mechanisms, the ubiquitin–proteasome system (UPS) is the major pathway responsible for selective protein degradation and plays essential roles in regulating cell proliferation, apoptosis, inflammation, and disease progression [11]. The ubiquitination process is mediated by a sequential enzymatic cascade involving ubiquitin-activating enzymes (E1), ubiquitin-conjugating enzymes (E2), and ubiquitin ligases (E3), among which E3 ligases determine substrate specificity by recognizing target proteins [12]. Dysregulation of UPS-mediated protein degradation has been implicated in multiple diseases, including cancer, inflammatory disorders, and reproductive system diseases [13]. In addition to E3 ligases themselves, substrate-associated regulatory proteins can also influence ubiquitination efficiency by modulating the interaction between E3 ligases and target proteins. Such competitive binding mechanisms have emerged as an important mode of regulating protein stability [14]. Based on this mode of regulation, we investigated whether the accumulation of Cav1.3 in EMs results from altered ubiquitin-dependent degradation and whether Cav1.3-interacting proteins affect its recognition by a specific E3 ligase.

Our preliminary observations showed that collapsin response mediator protein 4 (CRMP4) was elevated in endometriotic stromal cells, raising the possibility that CRMP4 participates in the regulation of Cav1.3 stability. We therefore examined the relationship among CRMP4, Cav1.3, and the E3 ubiquitin ligase RNF138. Because the increased abundance of CRMP4 also suggested altered transcriptional regulation, we further focused on zinc finger protein 460 (ZNF460), a zinc finger transcription factor involved in cell proliferation, tumor progression, and cellular adaptation [15], as a potential upstream regulator of CRMP4. In this study, we investigated whether ZNF460-dependent CRMP4 expression interferes with RNF138-mediated ubiquitination and degradation of Cav1.3 and assessed the role of this regulatory pathway in apoptosis resistance and the progression of EMs using cellular and mouse models.

## 2. Results

### 2.1. Impaired Ubiquitin–Proteasome Degradation Drives Cav1.3 Protein Accumulation in hEM15A Cells

**Figure 1:**
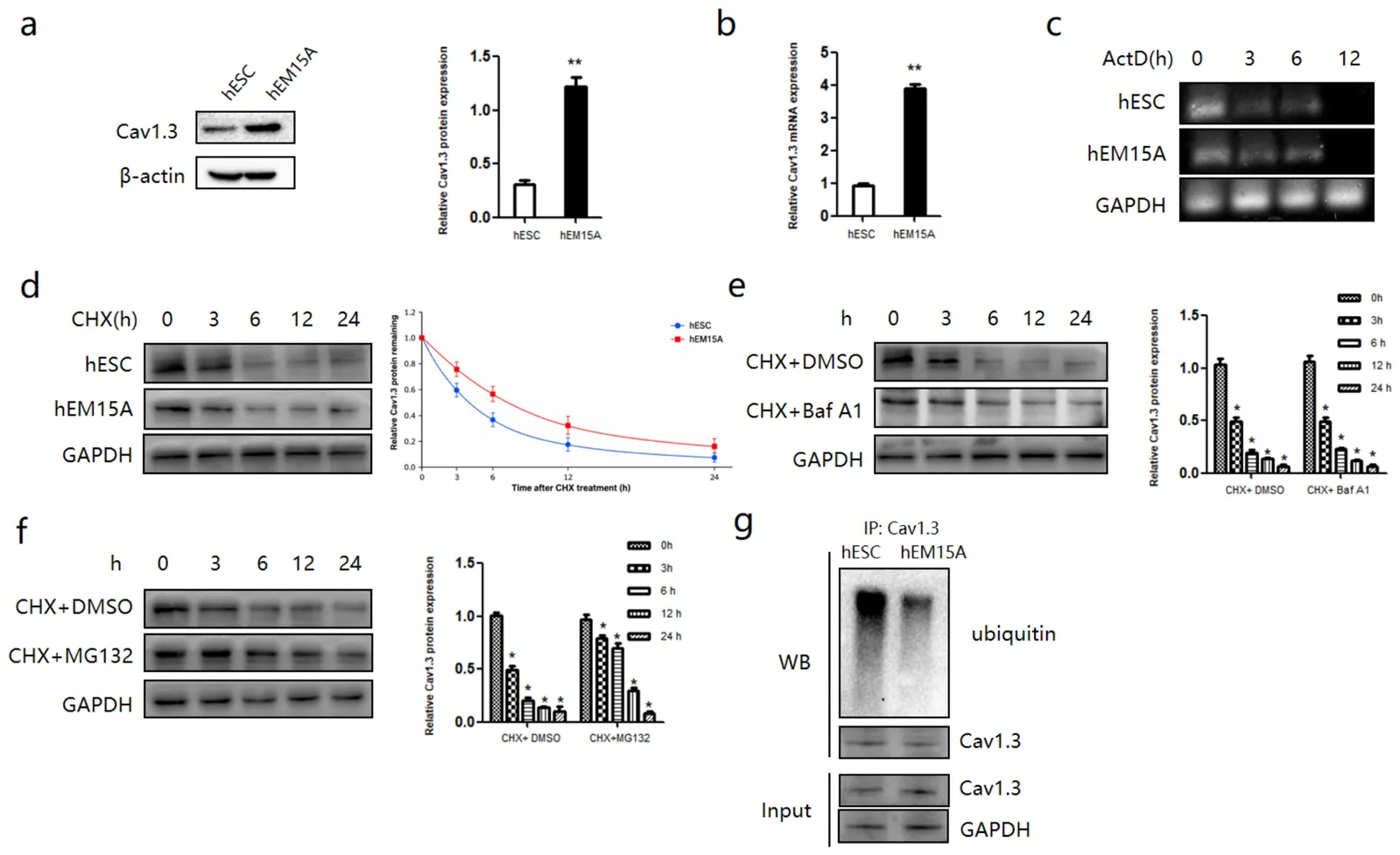
Cav1.3 is upregulated in hEM15A cells owing to impaired ubiquitin–proteasome-mediated degradation. (**a**) Representative Western blot analysis and quantitative densitometric analysis showing that Cav1.3 protein expression was significantly increased in hEM15A cells compared with hESCs. β-actin served as the loading control. (**b**) RT-qPCR analysis demonstrating that Cav1.3 mRNA expression was significantly elevated in hEM15A cells relative to hESCs. (**c**) Following treatment with actinomycin D (ActD), the degradation rate of Cav1.3 mRNA was comparable between hESCs and hEM15A cells, indicating that the increased Cav1.3 expression was not attributable to altered mRNA stability. (**d**) Representative Western blots and Cav1.3 degradation curves following CHX treatment in hESCs and hEM15A cells. Fitted lines show one-phase exponential decay models used to estimate protein half-lives. (**e**) Treatment with the lysosomal inhibitor bafilomycin A1 (BafA1) failed to rescue CHX-induced Cav1.3 degradation, suggesting that lysosomal degradation is not the major pathway regulating Cav1.3 turnover. (**f**) Treatment with the proteasome inhibitor MG132 significantly attenuated CHX-induced Cav1.3 degradation, indicating that Cav1.3 is predominantly degraded through the ubiquitin–proteasome pathway. (**g**) Immunoprecipitation of endogenous Cav1.3 followed by immunoblotting with an anti-ubiquitin antibody demonstrated that ubiquitination of Cav1.3 was markedly reduced in hEM15A cells compared with hESCs. Data are presented as the mean ± SD from three independent biological replicates (*n* = 3). * *p* < 0.05, ** *p* < 0.01.

To clarify the expression changes of Cav1.3 in endometriosis, we first examined the expression levels of Cav1.3 in hESC and hEM15A cells. The results of qPCR and Western blot showed that the mRNA and protein expressions of Cav1.3 in hEM15A cells were significantly higher than those in hESC (Figure 1a,b). To further clarify the reason for the increased expression of Cav1.3, we first examined the stability of its mRNA. After treatment with Actinomycin D (ActD), there was no significant difference in the degradation rate of Cav1.3 mRNA in both groups of cells (Figure 1c), suggesting that mRNA stability was not the main reason for the increase in its expression. Subsequently, we used cycloheximide (CHX) to inhibit protein synthesis and monitored Cav1.3 protein degradation over time. The normalized degradation curves showed slower Cav1.3 turnover in hEM15A cells than in hESCs. Fitting with a one-phase exponential decay model yielded estimated Cav1.3 half-lives of 3 h in hESCs and 6 h in hEM15A cells (Figure 1d), supporting increased Cav1.3 protein stability in hEM15A cells. To further clarify its degradation pathway, we treated the cells with proteasome inhibitor MG132 and lysosome inhibitor Bafilomycin A1 (BafA1). MG132 could significantly increase the protein level of Cav1.3, while no significant change was observed after BafA1 treatment (Figure 1e,f), suggesting that Cav1.3 is mainly degraded through the ubiquitin–proteasome pathway. Further immunoprecipitation was used to detect the ubiquitination level of Cav1.3, and the results showed that the ubiquitination modification of Cav1.3 in hEM15A cells was significantly lower than that in hESC (Figure 1g). Cav1.3 upregulation in hEM15A cells involves increased mRNA abundance and enhanced protein stability. The present study specifically identifies impaired ubiquitin–proteasome degradation as an additional post-translational mechanism contributing to Cav1.3 accumulation.

### 2.2. RNF138-Mediated Cav1.3 Ubiquitination Is Impaired in hEM15A Cells

**Figure 2:**
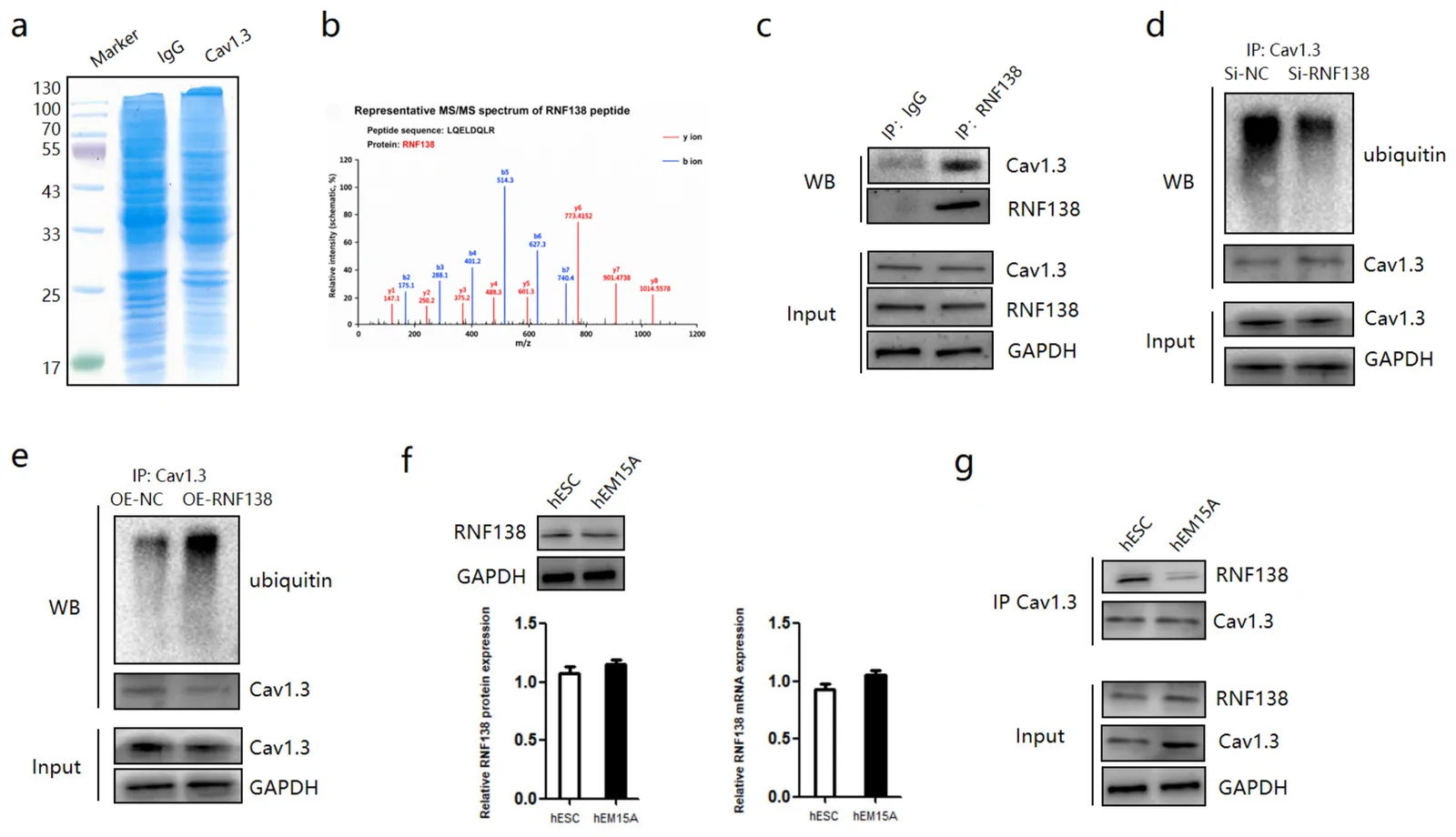
Identification of RNF138 as a Cav1.3-interacting E3 ubiquitin ligase regulating Cav1.3 ubiquitination. (**a**) Cav1.3-interacting proteins were identified by immunoprecipitation coupled with LC-MS/MS analysis. Cav1.3 was immunoprecipitated from hEM15A cells, followed by Coomassie Brilliant Blue staining and LC-MS/MS identification of candidate binding proteins. (**b**) Representative MS/MS spectrum of an identified RNF138-derived peptide. The peptide sequence and corresponding fragment ions confirmed the presence of RNF138 in Cav1.3 immunoprecipitates. (**c**) Validation of the interaction between RNF138 and Cav1.3 by endogenous co-immunoprecipitation. RNF138 was immunoprecipitated, and the associated Cav1.3 protein was detected by western blotting. (**d**) Knockdown of RNF138 reduces Cav1.3 ubiquitination. hEM15A cells were transfected with RNF138 siRNA or control siRNA, followed by Cav1.3 immunoprecipitation and ubiquitin immunoblotting. (**e**) Overexpression of RNF138 enhances Cav1.3 ubiquitination. hEM15A cells were transfected with RNF138 overexpression plasmid or control vector, and Cav1.3 ubiquitination levels were analyzed by immunoprecipitation followed by western blotting. (**f**) Expression analysis of RNF138 in hESC and hEM15A cells. RNF138 protein and mRNA levels were detected by western blotting and RT-qPCR, respectively. (**g**) Reduced interaction between RNF138 and Cav1.3 in hEM15A cells. Endogenous Cav1.3 was immunoprecipitated from hESC and hEM15A cells, and the associated RNF138 protein was detected by western blotting. Data are presented as the mean ± SD from three independent biological replicates (*n* = 3).

We further searched for the E3 ubiquitin ligases that mediate its ubiquitination degradation. Therefore, we performed co-immunoprecipitation using Cav1.3 as the bait protein and then cut the gel after Coomassie blue staining (Figure 2a). We identified the binding proteins using LC–MS/MS and further predicted the potential E3 ubiquitin ligases of Cav1.3 by combining the UbiBrowser database (http://ubibrowser.bio-it.cn/ubibrowser/home/index), and cross-screened the results with the mass spectrometry results. Based on the result, we finally selected RNF138 as the subsequent research object (Figure 2b). Subsequently, we further verified the interaction between RNF138 and Cav1.3 by Co-IP, and the results showed that RNF138 could form a stable protein complex with Cav1.3 (Figure 2c). To clarify whether RNF138 is involved in the regulation of Cav1.3 protein stability, we knocked down and overexpressed RNF138, respectively. RNF138 knockdown reduced the Cav1.3-associated ubiquitination signal, supporting the involvement of RNF138 in regulating Cav1.3 ubiquitination (Figure 2d). Conversely, RNF138 overexpression increased Cav1.3 ubiquitination and reduced its protein abundance (Figure 2e). Further detection of the expression of RNF138 in hESC and hEM15A cells revealed that there were no significant differences in mRNA or protein levels (Figure 2f). However, the Co-IP results showed that compared with hESC, the binding between RNF138 and Cav1.3 in hEM15A cells was significantly weakened (Figure 2g). These results support the involvement of RNF138 in regulating Cav1.3 ubiquitination and protein abundance. The reduced RNF138-Cav1.3 association in hEM15A cells, despite unchanged RNF138 expression, suggests that additional factors may modulate their association.

### 2.3. CRMP4 Reduces RNF138–Cav1.3 Association and Cav1.3 Ubiquitination

**Figure 3:**
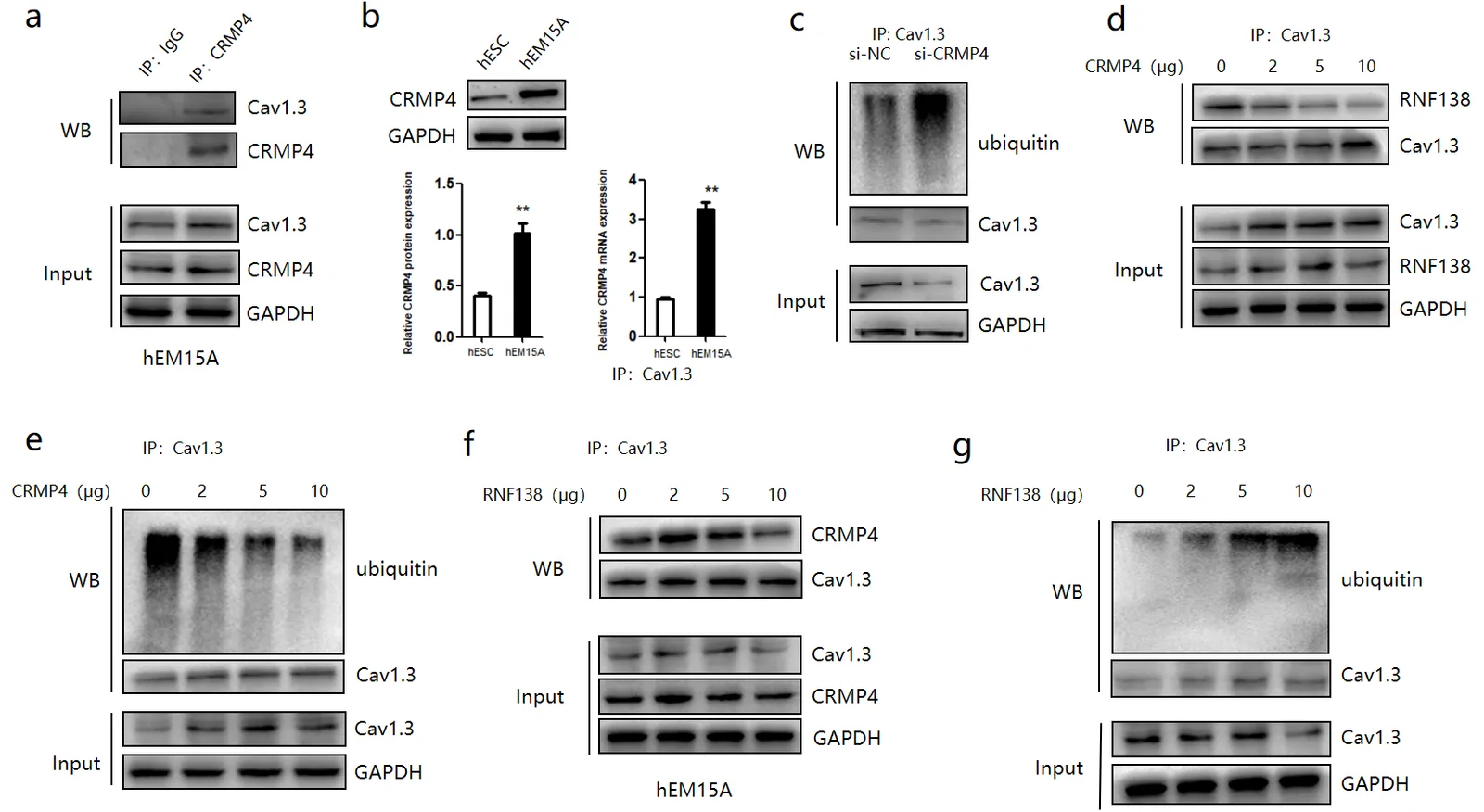
CRMP4 reduces RNF138-Cav1.3 association and Cav1.3-associated ubiquitination. (**a**) Validation of the interaction between CRMP4 and Cav1.3 by endogenous co-immunoprecipitation assay in hEM15A cells. Cav1.3 was immunoprecipitated, and the associated CRMP4 protein was detected by western blotting. (**b**) Expression analysis of CRMP4 in hESC and hEM15A cells. CRMP4 protein and mRNA levels were determined by western blotting and RT-qPCR, respectively. (**c**) Knockdown of CRMP4 enhances Cav1.3 ubiquitination. hEM15A cells were transfected with CRMP4 siRNA or control siRNA, followed by Cav1.3 immunoprecipitation and ubiquitin immunoblotting. (**d**) Increasing CRMP4 expression reduces the interaction between RNF138 and Cav1.3. hEM15A cells were transfected with increasing amounts of CRMP4 plasmid, followed by Cav1.3 immunoprecipitation and detection of RNF138 binding. (**e**) CRMP4 overexpression decreases Cav1.3 ubiquitination in a dose-dependent manner. hEM15A cells were transfected with increasing amounts of CRMP4 plasmid, and Cav1.3 ubiquitination levels were analyzed by immunoprecipitation followed by western blotting. (**f**) Increasing RNF138 expression weakens the interaction between CRMP4 and Cav1.3. hEM15A cells were transfected with increasing amounts of RNF138 plasmid, followed by Cav1.3 immunoprecipitation and detection of CRMP4 binding. (**g**) RNF138 overexpression restores Cav1.3 ubiquitination despite CRMP4-mediated protection. hEM15A cells were transfected with increasing amounts of RNF138 plasmid, and Cav1.3 ubiquitination levels were examined by immunoprecipitation followed by western blotting. Data are presented as the mean ± SD from three independent biological replicates (*n* = 3). ** *p* < 0.01.

Because RNF138 expression remained unchanged while its association with Cav1.3 was reduced, we investigated whether other Cav1.3-associated proteins could modulate RNF138-Cav1.3 association and Cav1.3 ubiquitination in hEM15A cells. To verify this hypothesis, we further analyzed the candidate interacting proteins identified by LC–MS/MS, screened the candidate protein CRMP4. Subsequently, we used Co-IP to further verify the interaction between CRMP4 and Cav1.3, and the results showed that CRMP4 could form a stable protein complex with Cav1.3 (Figure 3a). Further detection of the expression of CRMP4 in hESC and hEM15A cells revealed that compared with hESC, the mRNA and protein levels of CRMP4 were significantly increased in hEM15A cells (Figure 3b), suggesting that CRMP4 might be involved in the regulation of abnormal accumulation of Cav1.3 in EMs. To further clarify the effect of CRMP4 on the stability of Cav1.3 protein, we knocked down CRMP4 in hEM15A cells, and the results showed that the ubiquitination level of Cav1.3 significantly increased, while the protein expression of Cav1.3 significantly decreased (Figure 3c), indicating that CRMP4 can negatively regulate the ubiquitination degradation of Cav1.3. Given the effects of RNF138 perturbation on Cav1.3 ubiquitination, we further examined whether changes in CRMP4 expression affected RNF138–Cav1.3 association. Co-IP results showed that as the overexpression level of CRMP4 gradually increased, the binding between RNF138 and Cav1.3 gradually weakened, while the protein expression of Cav1.3 gradually increased in a dose-dependent manner (Figure 3d,e). Conversely, after overexpressing RNF138, the binding between CRMP4 and Cav1.3 significantly weakened, while the ubiquitination level of Cav1.3 significantly increased and the protein expression significantly decreased, also in a dose-dependent manner (Figure 3f,g). These findings support a role for CRMP4 in reducing RNF138–Cav1.3 association and Cav1.3-associated ubiquitination, without establishing direct competition for the same binding site.

### 2.4. ZNF460 Activates CRMP4 Transcription

**Figure 4:**
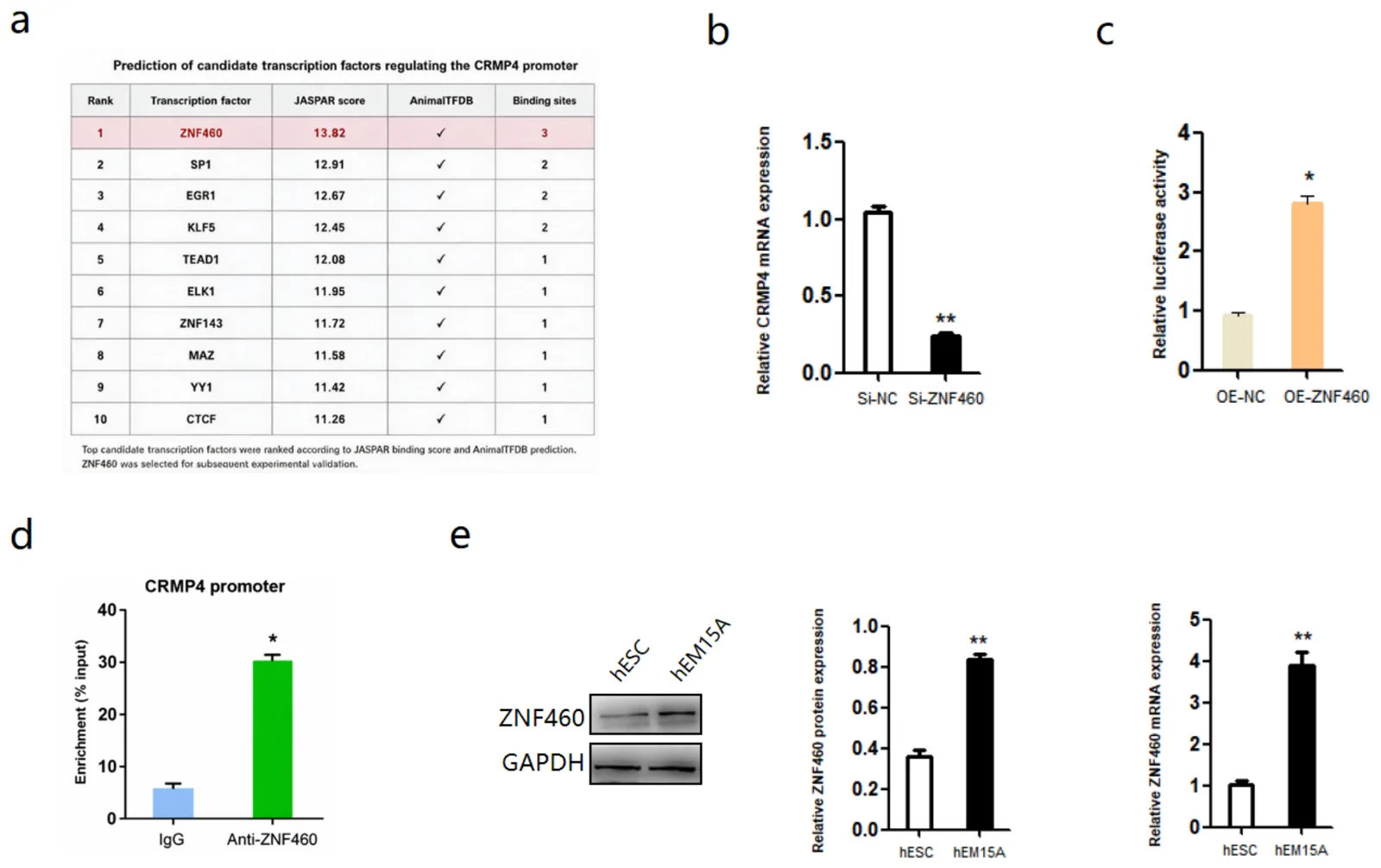
ZNF460 transcriptionally activates CRMP4 expression in endometriosis stromal cells. (**a**) Prediction of candidate transcription factors binding to the CRMP4 promoter. Potential transcription factors were screened using the JASPAR databases. The top 10 candidate transcription factors were ranked according to predicted binding scores and binding site numbers, and ZNF460 was selected for further investigation. (**b**) Effect of ZNF460 knockdown on CRMP4 expression. hEM15A cells were transfected with ZNF460 siRNA or control siRNA, and CRMP4 mRNA expression was analyzed by RT-qPCR. (**c**) ZNF460 promotes CRMP4 promoter activity. hEM15A cells were co-transfected with CRMP4 promoter luciferase reporter plasmid and ZNF460 overexpression plasmid or control vector, and relative luciferase activity was measured. (**d**) ChIP–qPCR analysis of ZNF460 enrichment at the CRMP4 promoter in hEM15A cells. Chromatin was immunoprecipitated using an anti-ZNF460 antibody, with IgG serving as the negative control. The recovered CRMP4 promoter DNA was quantified by qPCR and expressed as a percentage of input. (**e**) Expression analysis of ZNF460 in hESC and hEM15A cells. ZNF460 protein and mRNA levels were determined by western blotting and RT-qPCR, respectively. Data are presented as the mean ± SD from three independent biological replicates (*n* = 3). * *p* < 0.05, ** *p* < 0.01.

Given that the aforementioned results confirmed the abnormal high expression of CRMP4 in hEM15A cells, we further explored its upstream transcriptional regulatory mechanism. Firstly, using the JASPAR databases, we predicted the potential binding transcription factors in the CRMP4 promoter region, and selected 10 candidate transcription factors based on the predicted scores and the number of binding sites (Figure 4a). Subsequently, we knocked down each candidate transcription factor using siRNA and detected CRMP4 expression. The results showed that only knocking down ZNF460 could significantly reduce CRMP4 mRNA expression, so ZNF460 was chosen for further study (Figure 4b). Subsequently, we used the dual luciferase reporter gene assay to detect the effect of ZNF460 on the activity of the CRMP4 promoter. The results showed that after overexpression of ZNF460, the activity of the CRMP4 promoter was significantly enhanced (Figure 4c). Further, we verified the binding relationship between ZNF460 and the CRMP4 promoter through ChIP-qPCR experiments, and the results confirmed that ZNF460 could directly bind to the CRMP4 promoter region (Figure 4d). Finally, we detected the expression of ZNF460 in hESC and hEM15A cells, and RT-qPCR and Western blot results showed that the mRNA and protein expression of ZNF460 in hEM15A cells were significantly higher than those in hESC (Figure 4e). In conclusion, ZNF460 can directly bind to the CRMP4 promoter and activate its transcription, and is an important upstream regulatory factor leading to the abnormal high expression of CRMP4 in hEM15A cells.

### 2.5. ZNF460 Regulates the Stability of Cav1.3 Through CRMP4 and Inhibits Apoptosis in hEM15A Cells

**Figure 5:**
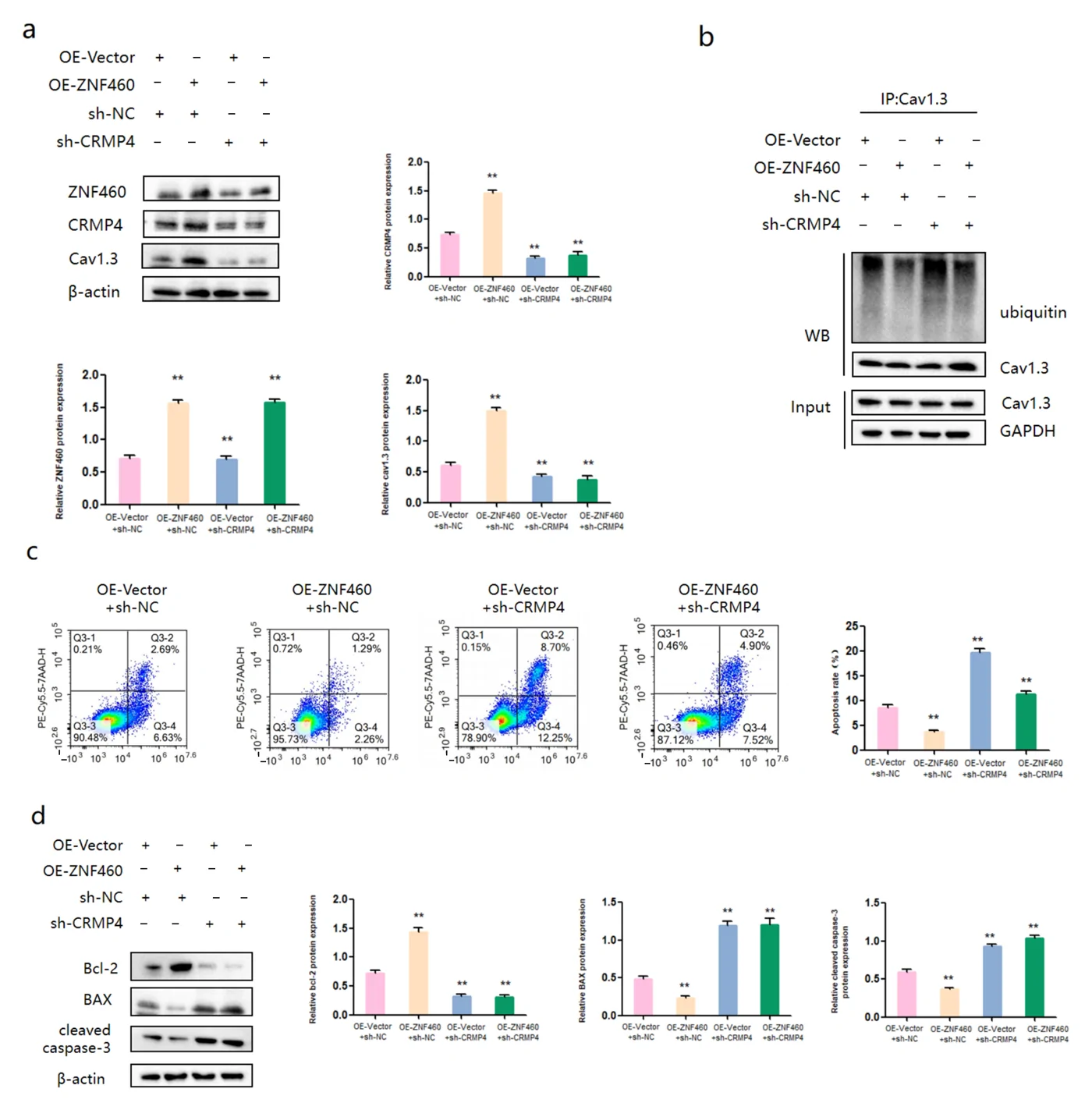
Effects of ZNF460 overexpression and CRMP4 knockdown on Cav1.3 protein expression, ubiquitination, and cell apoptosis. The hEM15A were assigned to four groups: OE-Vector + sh-NC, OE-ZNF460 + sh-NC, OE-Vector + sh-CRMP4, and OE-ZNF460 + sh-CRMP4. (**a**) Representative Western blots and densitometric analyses of ZNF460, CRMP4, and Cav1.3 protein expression. (**b**) Immunoprecipitation of Cav1.3 followed by immunoblotting with anti-ubiquitin and anti-Cav1.3 antibodies to assess Cav1.3-associated ubiquitination. Input lysates were immunoblotted for Cav1.3 and GAPDH. (**c**) Representative flow cytometry plots and quantification of apoptosis following Annexin V–APC/7-AAD staining. (**d**) Representative Western blots and densitometric analyses of Bcl-2, BAX, and cleaved caspase-3 protein expression. Data are presented as mean ± SD from 3 independent experiments using separately prepared cell cultures. Statistical significance was assessed using two-way ANOVA with ZNF460 overexpression and CRMP4 knockdown as factors, followed by Tukey’s multiple-comparison test. Colors in the flow cytometry plots indicate event density, increasing from blue to green, yellow, and red. ** *p* < 0.01.

To determine whether CRMP4 mediates the effects of ZNF460 on Cav1.3 protein stability and apoptosis resistance, hEM15A cells were assigned to four groups: OE-Vector + sh-NC, OE-ZNF460 + sh-NC, OE-Vector + sh-CRMP4, and OE-ZNF460 + sh-CRMP4. Western blotting showed that, compared with the OE-Vector + sh-NC group, ZNF460 overexpression increased CRMP4 and Cav1.3 protein levels. In contrast, CRMP4 knockdown alone reduced Cav1.3 protein abundance. Importantly, CRMP4 knockdown markedly attenuated the increase in Cav1.3 protein induced by ZNF460 overexpression, despite the sustained overexpression of ZNF460 in the OE-ZNF460 + sh-CRMP4 group (Figure 5a). Cav1.3 was subsequently immunoprecipitated, followed by immunoblotting with an anti-ubiquitin antibody. ZNF460 overexpression reduced the ubiquitination level of Cav1.3, whereas CRMP4 knockdown alone increased Cav1.3 ubiquitination. Moreover, the reduction in Cav1.3 ubiquitination caused by ZNF460 overexpression was substantially reversed by CRMP4 knockdown (Figure 5b). These findings indicate that CRMP4 mediates the inhibitory effect of ZNF460 on Cav1.3 ubiquitination, thereby contributing to Cav1.3 protein stabilization. Annexin V-APC/7-AAD staining followed by flow cytometry showed that ZNF460 overexpression decreased the apoptotic rate of hEM15A cells, whereas CRMP4 knockdown increased apoptosis. Compared with the OE-ZNF460 + sh-NC group, the OE-ZNF460 + sh-CRMP4 group exhibited a significantly higher apoptotic rate, indicating that CRMP4 knockdown attenuated the anti-apoptotic effect of ZNF460 overexpression (Figure 5c). Consistently, ZNF460 overexpression increased Bcl-2 expression and decreased the levels of BAX and cleaved caspase-3. CRMP4 knockdown produced the opposite changes and substantially reversed the alterations in these apoptosis-related proteins induced by ZNF460 overexpression (Figure 5d). These findings support the involvement of CRMP4 in the effects of ZNF460 on Cav1.3 protein stability and apoptosis resistance in hEM15A cells. ZNF460-induced CRMP4 expression contributes to reduced Cav1.3 ubiquitination and increased protein abundance, accompanied by reduced apoptosis in hEM15A cells.

### 2.6. ZNF460 Promotes the Growth of Endometriosis Lesions and Inhibits Cell Apoptosis

**Figure 6:**
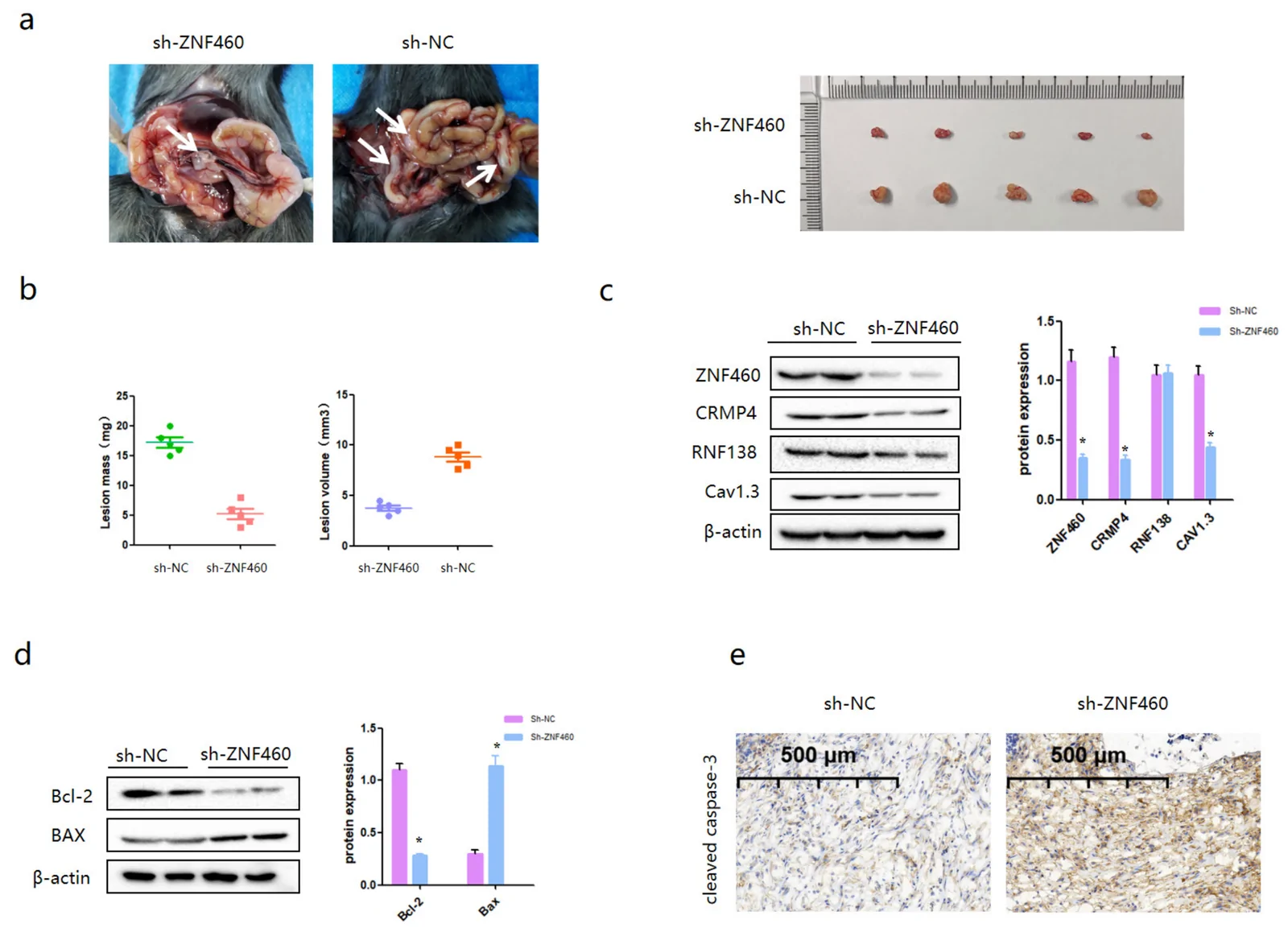
Effects of ZNF460 knockdown on lesion growth and apoptosis-associated protein expression in vivo. (**a**) Representative gross images of lesions in situ (**left**) and excised lesions (**right**) from the sh-ZNF460 and sh-NC groups. White arrows indicate lesions. (**b**) Mass and volume of excised lesions. (**c**) Representative Western blots and densitometric analyses of ZNF460, CRMP4, RNF138, and Cav1.3 in lesion tissues. (**d**) Representative Western blots and densitometric analyses of Bcl-2 and BAX in lesion tissues. (**e**) Representative immunohistochemical staining for cleaved caspase-3 in lesion tissues. Brown staining indicates immunoreactivity. Data are presented as mean ± SD; *n* = 5 mice per group. * *p* < 0.05.

To examine the effects of ZNF460 silencing on lesion development and CRMP4/Cav1.3 expression in vivo, we established a mouse endometriosis model using uterine tissues from AAV-treated donor mice. After the modeling was completed, the ectopic lesions were observed and measured. The results showed that compared with the control group, the volume and weight of the ectopic lesions in mice were significantly reduced after ZNF460 silencing (Figure 6a,b), suggesting that ZNF460 promotes the growth of ectopic lesions. Western blotting of ectopic lesion tissues confirmed a reduction in ZNF460 protein expression following AAV-shZNF460 treatment. This reduction was accompanied by decreased CRMP4 and Cav1.3 protein levels, whereas RNF138 protein expression remained largely unchanged (Figure 6c). These changes were consistent with the molecular effects observed in hEM15A cells and support an association between ZNF460 expression and the CRMP4/Cav1.3 pathway in vivo. Consistently, Western blotting showed increased BAX protein levels and reduced Bcl-2 expression in the AAV-shZNF460 group (Figure 6d). Apoptosis within the ectopic lesions was subsequently evaluated by immunohistochemical staining for cleaved caspase-3. Representative immunohistochemical images showed more prominent cleaved caspase-3 staining in lesions from the AAV-shZNF460 group than in those from the AAV-shNC group. (Figure 6e), indicating increased apoptosis in ectopic lesion tissues following ZNF460 silencing. These results show that ZNF460 silencing in donor mice before transplantation reduces subsequent lesion growth and increases lesion-associated apoptosis, accompanied by decreased CRMP4 and Cav1.3 expression.

## 3. Discussion

Resistance to apoptosis allows ectopic endometrial cells to survive after entering the peritoneal cavity and contributes to lesion establishment and persistence [16,17]. The present results link this survival advantage to the regulation of Cav1.3 protein turnover. ZNF460 promoted CRMP4 transcription, while increased CRMP4 was associated with reduced RNF138–Cav1.3 interaction, decreased Cav1.3 ubiquitination, and prolonged Cav1.3 stability. Together with the cell experiments and animal experiments, these results support a model in which CRMP4 connects transcriptional dysregulation to Cav1.3 accumulation and apoptosis resistance. However, the molecular basis of the reduced RNF138–Cav1.3 association remains unresolved. In particular, the current evidence does not fully distinguish direct competition for Cav1.3 binding from changes in channel trafficking or accessibility to RNF138.

Abnormal calcium signaling has been implicated in the proliferation, invasion, inflammatory responses, and survival of endometriotic cells [18]. Cav1.3 is a voltage-dependent L-type calcium channel [19], and its contribution to cell survival and proliferation has also been reported in cancer models [20,21]. Our previous work linked Cav1.3 to reduced Caspase-3 activation and apoptosis in endometriotic stromal cells. Here, the cycloheximide chase and MG132 experiments support a contribution of proteasome-dependent turnover to Cav1.3 abundance, whereas altered mRNA stability did not explain its accumulation. This distinction does not exclude additional regulation at the transcriptional or translational level. Moreover, increased total Cav1.3 protein should not be equated with increased functional channels at the plasma membrane. Determining whether the stabilized protein reaches the cell surface and increases calcium entry is necessary to connect altered protein turnover more directly to the anti-apoptotic phenotype.

A particularly informative result was the reduction in RNF138–Cav1.3 association despite no significant change in RNF138 expression in hEM15A cells. E3 ubiquitin ligases contribute substrate specificity to the ubiquitination machinery [22], but their total abundance does not necessarily reflect their access to a particular substrate. The discrepancy observed here could therefore arise from altered localization of RNF138 or Cav1.3, changes in the conformation or modification of either protein, or recruitment of another component into the complex. Increased CRMP4 provides one plausible explanation because it is associated with Cav1.3 and reduced the association of RNF138 with the channel. Nevertheless, reduced co-immunoprecipitation indicates a change in recoverable association under the assay conditions; it does not, by itself, establish lower intrinsic binding affinity. The data therefore favor altered substrate engagement over reduced ligase expression, while leaving the underlying physical mechanism open.

CRMP4 has established roles in neuronal development, axonal guidance, cytoskeletal remodeling, and cancer progression [23,24]. Its relationship with the cytoskeleton raises the possibility that it stabilizes Cav1.3 partly by altering channel transport or retention within specific cellular compartments. Because Cav1.3 is a membrane protein and CRMP4 is predominantly associated with the cytoplasmic compartment, a direct interaction would be expected to involve a cytoplasm-facing region of Cav1.3. Such an interaction could occur at the plasma membrane or on intracellular membranes during channel biosynthesis, trafficking, or internalization. CRMP4 might therefore reduce Cav1.3 exposure to RNF138 without occupying the same binding site. Alternatively, CRMP4 binding could mask an RNF138-accessible region or induce a conformational change that makes ubiquitination less favorable. These possibilities would have different consequences for channel distribution and function, despite producing similar changes in total Cav1.3 abundance.

The molecular basis of CRMP4-dependent interference with RNF138–Cav1.3 association remains unresolved. The co-IP and dose-dependent experiments do not distinguish direct competition for an overlapping binding site from indirect effects on protein localization, conformation, or complex composition. It remains unknown whether CRMP4 and RNF138 recognize overlapping sequences within a Cav1.3 cytoplasmic domain, bind distinct regions that influence one another, or associate through additional proteins. Domain mapping and binding-deficient mutants would help distinguish these explanations, particularly if their effects on ubiquitination and protein turnover were assessed alongside their effects on localization. Co-immunoprecipitation also requires careful interpretation for a large multipass membrane protein such as Cav1.3. Extraction conditions can affect channel solubilization, complex recovery, and the preservation of full-length protein. Establishing that the recovered species represents intact Cav1.3, rather than a channel fragment, is therefore important for assigning the interaction to the full-length channel. Molecular-weight-resolved input and immunoprecipitation controls, antibodies recognizing distinct channel regions, and an independent interaction assay would strengthen this interpretation. Without such resolution, association within a complex should be distinguished from direct molecular contact.

The cellular stage at which Cav1.3 undergoes ubiquitination is another unresolved aspect of the proposed mechanism. Proteasome sensitivity does not identify whether RNF138 acts on newly synthesized Cav1.3 during endoplasmic reticulum quality control, on an intracellular trafficking pool, or on channels following membrane internalization. It also does not exclude a contribution from lysosomal turnover to other channel pools. CRMP4 could increase total Cav1.3 by protecting immature protein from degradation, prolonging surface residence, or redirecting internalized channels toward recycling. Distinguishing these possibilities will require compartment-specific measurements of channel maturation, surface abundance, and turnover. In addition, the RNF138-dependent ubiquitination results do not alone establish direct ubiquitin transfer from RNF138 to Cav1.3. Catalytic-dependence experiments and biochemical reconstitution would help resolve this point. Whether CRMP4 itself is an RNF138 substrate also warrants investigation: if so, changes in CRMP4 turnover could alter both the availability of RNF138 and the amount of CRMP4 associated with Cav1.3.

At the transcriptional level, promoter reporter assays and ChIP analysis support direct activation of CRMP4 by ZNF460. Previous studies have connected ZNF460 to gene expression programs involved in tumor progression and proliferation [25,26]. The rescue experiments place CRMP4 downstream of ZNF460 in the regulation of Cav1.3: CRMP4 depletion counteracted ZNF460-induced Cav1.3 accumulation, restored Cav1.3 ubiquitination, and reversed apoptosis suppression. This supports CRMP4 as a mediator of the measured effects but does not establish it as the only relevant ZNF460 target. Although CRMP4 depletion reversed both ZNF460-induced Cav1.3 accumulation and apoptosis suppression, these rescue experiments do not establish whether the anti-apoptotic effect requires Cav1.3. Cav1.3 knockdown in cells overexpressing ZNF460 or CRMP4 would be needed to distinguish its contribution from other downstream effects of this regulatory pathway. The signals responsible for ZNF460 upregulation also remain unidentified. Estrogen signaling, inflammatory cytokines, and hypoxia are candidate inputs for investigation, rather than established upstream components of this pathway.

Silencing ZNF460 in the mouse model reduced lesion growth, decreased CRMP4 and Cav1.3 expression, and increased lesion-associated apoptosis. These changes are consistent with the cellular findings but do not establish the extent to which the lesion phenotype depends on the CRMP4/RNF138/Cav1.3 pathway. ZNF460 may regulate additional genes affecting Cav1.3 expression or lesion growth, and its downstream targets were not comprehensively profiled in this study. Contributions from other survival pathways and lesion-associated cell populations therefore remain possible. Restoring CRMP4 or Cav1.3 expression following ZNF460 silencing would help determine the contribution of this pathway to the in vivo phenotype. The in vivo findings should be interpreted with caution because of the small sample size (*n* = 5 per group), the absence of investigator blinding, and the lack of specific measures to control potential confounding factors. These limitations may reduce the precision of the estimates and increase susceptibility to bias. The present study was conducted primarily in hEM15A cells, with primary hESCs serving as a reference for basal expression. Differences in immortalization status and culture conditions may influence protein expression and apoptotic responses, limiting the interpretation of comparisons between these models. The regulatory relationships identified here are supported by gene perturbation experiments within hEM15A cells; however, whether they are consistently observed in patient-derived ectopic stromal cells remains unknown. Further validation in primary cells from multiple patients and human endometriotic tissues is needed to assess the relevance of these findings across the heterogeneous forms of endometriosis. Although Cav1.3 protein stabilization was observed, total protein abundance does not establish increased plasma membrane localization or channel activity. Neither membrane-localized Cav1.3 nor calcium influx was assessed in this study. Therefore, whether altered Cav1.3 stability contributes to the observed anti-apoptotic phenotype through changes in calcium signaling remains unresolved. The central implication of the present work is that Cav1.3 accumulation can arise through altered access to the ubiquitination machinery even when RNF138 expression remains unchanged.

## 4. Materials and Methods

### 4.1. Cell Culture

Both the human immortalized eutopic endometrial stromal cell line 15A (hEM15A) and primary human endometrial stromal cells (hESCs) were purchased from the China Center for Type Culture Collection (CCTCC, Wuhan, China). hEM15A cells were cultured in RPMI-1640 medium (HyClone, Logan, UT, USA) supplemented with 15% fetal bovine serum (FBS; Gibco, Thermo Fisher Scientific, Waltham, MA, USA) under a humidified atmosphere containing 5% CO_2_ at 37 °C. hESCs were maintained in Dulbecco’s ModifiedEagle Medium/Nutrient Mixture F-12 (DMEM/F12, HyClone, Logan, UT, USA) supplemented with 10% FBS and 1% streptomycin (Solarbio, Beijing, China). All cells were incubated at 37 °C in a 5% CO_2_ humidified incubator. The culture medium was replaced with fresh medium routinely to maintain cell growth and viability.

### 4.2. Cell Transfection

The expression plasmids of CRMP4, RNF138 and ZNF460 were constructed by Shanghai Jima Gene Company in China. The shRNAs and siRNAs targeting CRMP4, RNF138 and ZNF460 were synthesized by Shanghai Jima Gene Company in China. The cells were transfected using Lipofectamine 3000 reagent (Invitrogen, Thermo Fisher Scientific, Carlsbad, CA, USA) according to the instructions, and the cells were collected 48 h after transfection for subsequent experiments.

### 4.3. RNA Extraction and Real-Time Fluorescence Quantitative PCR (qRT-PCR)

Total RNA was extracted from the cells using the TRIzol reagent (Invitrogen, Carlsbad, CA, USA) and immediately reverse transcribed into cDNA using the PrimeScript RT Master Mix (Takara, Shiga, Japan) according to the manufacturer’s instructions. Then, RT-qPCR was performed using SYBR Premix Ex Taq II (Takara, Shiga, Japan) on a CFX Duet Real-Time PCR System (Bio-Rad Laboratories, Hercules, CA, USA). With *GAPDH* as a reference, the mRNA expression level of the target gene was calculated using the 2^−ΔΔCt^ method. The primers were as follows:

Human: GAPDH(F: 5′-GGAGCGAGATCCCTCCAAAAT-3′;

R: 5′-GGCTGTTGTCATACTTCTCATGG-3′);

Cav1.3(F: 5′-CTTCGACAACGTCCTCTCTGCT-3′;

R: 5′-GCCGATGTTCTCTCCATTCGAG-3′);

RNF138(F: 5′-CCACGTCCTACACCGAAGAT-3′;

R: 5′-GCATCTGCAGCTACCAGAAA-3′);

CRMP4(F: 5′-GCTGGAAGAGATCGTGGTGA-3′;

R: 5′-CAGGATGGTGATGTTGGTGC-3′);

ZNF460(F: 5′-GAGGAGGAGATGGAGGAGGA-3′;

R: 5′-CTGCTGCTGCTGTTGTTGTT-3′).

### 4.4. Western Blot

Cells were collected and lysed. Total protein was extracted and quantified using the BCA Protein Assay Kit (Solarbio, Beijing, China), following the manufacturer’s instructions. Primary antibodies used included anti-Cav1.3 (1:1000; Abcam, Cambridge, UK; ab191038), anti-RNF138 (1:1000; Abcam; ab92730), anti-CRMP4 (1:1000; Abcam; ab126787), anti-ZNF460 (1:1000; Bioworld Technology, Nanjing, China; BS5980), anti-Bcl-2 (1:1000; Abcam; ab182858), anti-Bax (1:1000; Abcam; ab32503), anti-cleaved caspase-3 (1:1000; Abcam; ab2302), anti-GAPDH (1:5000; Abcam; ab8245), and anti-β-actin (1:5000; Abcam; ab8227) and were incubated at 4 °C overnight. After washing with tris-buffered saline with Tween (TBS-T) three times, the secondary antibody (Solarbio, Beijing, China) was added for 1 h at room temperature. The ECL substrate (Solarbio, Beijing, China) was used to detect the expression of target proteins in the FUSION FX5 imaging system (VILBER, Collégien, France).

### 4.5. Cycloheximide Chase Assay

Cells were treated with cycloheximide (CHX, 100 μg/mL) to inhibit protein synthesis and harvested at 0, 3, 6, 12, and 24 h. Cav1.3 protein abundance was assessed by Western blotting. For each independent experiment, Cav1.3 band intensities were normalized to the corresponding GAPDH signals and then expressed relative to the 0-h value within each cell model. The normalized data were plotted against treatment time and fitted using a one-phase exponential decay model in GraphPad Prism 9.0. Protein half-life (t_1_/_2_) was estimated from the fitted decay rate constant (k) using t_1_/_2_ = ln(2)/k.

### 4.6. Actinomycin D Experiment

Using Actinomycin D (5 μg/mL) to inhibit RNA transcription, cells were collected at different time points, RNA was extracted, and the expression changes of Cav1.3 and CRMP4 mRNA were detected to evaluate the stability of mRNA.

### 4.7. Combined Treatment with CHX and MG132 or Bafilomycin A1

Cells were treated with cycloheximide (CHX, 100 μg/mL) in combination with MG132 (10 μM), bafilomycin A1 (BafA1, 100 nM), or DMSO as the vehicle control. Cells were harvested at 0, 3, 6, 12, and 24 h after treatment, and Cav1.3 protein levels were analyzed by Western blotting to assess the contributions of proteasomal and lysosomal pathways to Cav1.3 degradation.

### 4.8. Immunoprecipitation and Ubiquitination Detection

Immunoprecipitation was performed using Protein A/G magnetic beads. Cell lysates were added with anti-Cav1.3 or RNF138 antibodies and incubated at 4 °C overnight. Then, Protein A/G magnetic beads were added for further incubation. After washing, Loading Buffer was added for boiling elusion, and Western blot was used to detect protein interactions. The ubiquitination experiment was conducted by immunoprecipitation with anti-Cav1.3 antibody, followed by detection of Cav1.3 ubiquitination level using anti-ubiquitin antibody.

### 4.9. Coomassie Brilliant Blue Staining and LC–MS/MS Analysis

After performing immunoprecipitation with anti-Cav1.3 antibody, the proteins were separated by SDS-PAGE and stained with Coomassie Brilliant Blue. The target bands were cut out and sent to Shanghai Baipu Biotechnology Co., Ltd. (Shanghai, China). for liquid chromatography–tandem mass spectrometry (LC–MS/MS) analysis. The protein identification results were annotated and analyzed in conjunction with the UniProt database.

### 4.10. UbiBrowser Prediction

Using the UbiBrowser database (http://ubibrowser.bio-it.cn), potential E3 ubiquitin ligases that may mediate the ubiquitination of Cav1.3 were predicted. Based on the predicted scores and literature reports, candidate E3 ligases were selected for subsequent experimental verification.

### 4.11. Prediction from the JASPAR Database

Using the JASPAR database, the potential binding sites of transcription factors in the promoter region of CRMP4 were analyzed. Based on the predicted scores, candidate transcription factors were selected, and the subsequent research subjects were determined by combining the results of siRNA screening.

### 4.12. Dual Luciferase Reporter Assay

The wild-type and mutant sequences of the CRMP4 promoter were each cloned into the pGL3 reporter vector and were co-transfected with the ZNF460 expression plasmid into hEM15A cells. After 48 h, the luciferase activity was determined using the dual-luciferase assay kit, and the relative luciferase activity was calculated.

### 4.13. Chromatin Immunoprecipitation Followed by Quantitative PCR (ChIP–qPCR)

ChIP was performed using a commercial kit (Beyotime Biotechnology, Shanghai, China) according to the manufacturer’s instructions. Cells were cross-linked with formaldehyde, and chromatin was fragmented by sonication. The sheared chromatin was immunoprecipitated with an anti-ZNF460 antibody, with matched IgG serving as the negative control. An aliquot of chromatin was retained as the input control. Following reversal of cross-links and DNA purification, the recovered DNA was analyzed by qPCR using primers targeting the CRMP4 promoter. Enrichment was calculated as a percentage of input, accounting for the dilution of the input sample.

### 4.14. Animals

Female C57BL/6 mice (6–8 weeks old) were purchased from the Experimental Animal Center of Lanzhou University Medical College (Lanzhou, China). Donor mice received tail-vein injections of AAV9 vectors expressing shRNA targeting mouse ZNF460 (AAV9-shZNF460) or a non-targeting control shRNA (AAV9-shNC) on experimental days 1 and 7. Each injection contained 10 μL of viral stock at a titer of 1 × 10^13^ vg/mL, corresponding to 1 × 10^11^ vg per mouse per administration, diluted with 200 μL of saline. On day 14, uterine tissues were collected, minced into approximately 1 mm^3^ fragments, and transplanted into recipient mice by intraperitoneal injection to establish the endometriosis model. Recipient mice were euthanized on experimental day 28, corresponding to 14 days after uterine tissue transplantation, and ectopic lesions were collected for subsequent analyses. The lesion tissues were collected for Western blot, immunohistochemistry detection. Lesion length and width were measured using a vernier caliper, and lesion volume was calculated as V = 0.52 × length × width^2^, with dimensions measured in millimeters and volume expressed in mm^3^. All animal experiments were approved by the First Hospital of Lanzhou University Research Ethics Committee (approval No. LDYYSZ2025-09).

### 4.15. Flow Cytometry Analysis

The experiment was conducted using the Annexin V-APC/7-AAD cell apoptosis detection kit (Solarbio, Beijing, China) according to the instructions. After transfection for 48 h, the cells were collected and washed with PBS, then resuspended in binding buffer. Annexin V-APC/7-AAD staining solutions were added successively, and the cells were incubated in the dark for 15 min. The apoptosis of the cells was detected by flow cytometry, and the proportions of early and late apoptotic cells were analyzed using FlowJo software (version 10.8.1).

### 4.16. Immunohistochemistry

Paraffin-embedded tissue sections were deparaffinized, rehydrated, and subjected to heat-induced antigen retrieval. After blocking endogenous peroxidase activity, sections were incubated overnight with an anti-cleaved caspase-3 antibody (1:500), followed by incubation with a secondary antibody (Solarbio, Beijing, China). Immunoreactivity was visualized using 3,3′-diaminobenzidine (DAB), and sections were examined and photographed under a microscope.

### 4.17. Statistical Analysis

Data are presented as mean ± standard deviation (SD). The exact sample size (*n*), replicate type, and statistical test are specified in each figure legend. For cell-based assays, *n* denotes the number of independent experiments performed using separately prepared cell cultures; for animal experiments, *n* denotes the number of individual mice. Where technical replicates were used, their handling is described in the corresponding figure legend. Comparisons between two groups were performed using Student’s *t*-test. Multiple-group comparisons involving a single factor were analyzed using one-way ANOVA followed by Tukey’s multiple-comparison test. The factorial experiments in Statistical analyses were performed using GraphPad Prism 9.0 (GraphPad Software). *p* < 0.05 was considered statistically significant.

## 5. Conclusions

In summary, this study identifies a mechanism regulating Cav1.3 protein homeostasis in hEM15A cells. ZNF460 activates CRMP4 transcription, and the resulting increase in CRMP4 expression interferes with RNF138–Cav1.3 association, contributing to reduced Cav1.3 ubiquitination and increased protein stability. Changes in Cav1.3 protein homeostasis were associated with altered apoptotic responses in hEM15A cells. However, the present findings do not establish whether Cav1.3 is required for the anti-apoptotic effects of ZNF460/CRMP4. These findings provide a basis for further investigation of the ZNF460–CRMP4–Cav1.3 pathway in endometriosis. Its relevance across heterogeneous human lesions and its potential therapeutic implications require validation in patient-derived ectopic stromal cells and human endometriotic tissues.

## Data Availability

The data supporting the findings of this study are included in the article and Supplementary Materials. The raw LC-MS/MS data and protein identification results are available from the corresponding author upon reasonable request.

## Supplementary Materials

The following supporting information can be downloaded at: https://www.mdpi.com/article/10.3390/ijms27209291/s1.

## Abbreviations

AAV	adeno-associated virus
ANOVA	analysis of variance
BafA1	Bafilomycin A1
CHX	cycloheximide
Co-IP	co-immunoprecipitation
CRMP4	collapsin response mediator protein 4
EMs	endometriosis
hESCs	human endometrial stromal cells
hEM15A	human endometrial stromal cell line 15A
LC–MS/MS	liquid chromatography–tandem mass spectrometry
MG132	carbobenzoxy-Leu-Leu-leucinal
qRT-PCR	quantitative real-time reverse transcription polymerase chain reaction
RNF138	RING finger ubiquitin ligase 138
UPS	ubiquitin–proteasome system
ZNF460	zinc finger protein 460
